# Supplementary material for: 5′ modifications to CRISPR–Cas9 gRNA can change the dynamics and size of R-loops and inhibit DNA cleavage
Source: Nucleic Acids Res. 2020 Jun 4;48(12):6811–23. doi: 10.1093/nar/gkaa477 (PMC7337959; doi:10.1093/nar/gkaa477)
Supplement: gkaa477_Supplemental_File [file gkaa477_supplemental_file.pdf]

Supplementary Figures to:

5′ modifications to CRISPR Cas9 gRNA can change the dynamics and size of R-loops and inhibit DNA cleavage

Grace Mullally[1,2], Kara van Aelst[1], Mohsin M. Naqvi[1], Fiona M. Diffin[1], Tautvydas Karvelis[3], Giedrius Gasiunas[3,4], Virginijus Siksnys[3] and Mark D. Szczelkun[1,2]

[1]DNA-Protein Interactions Unit, School of Biochemistry, University of Bristol, BS8 1TD, UK

[2]BrisSynBio, Life Sciences Building, Tyndall Avenue, University of Bristol, Bristol, BS8 1TQ, UK

[3]Institute of Biotechnology, Vilnius University, Vilnius, Lithuania

[4]CasZyme, Saulėtekio al. 7A, Vilnius, Lithuania

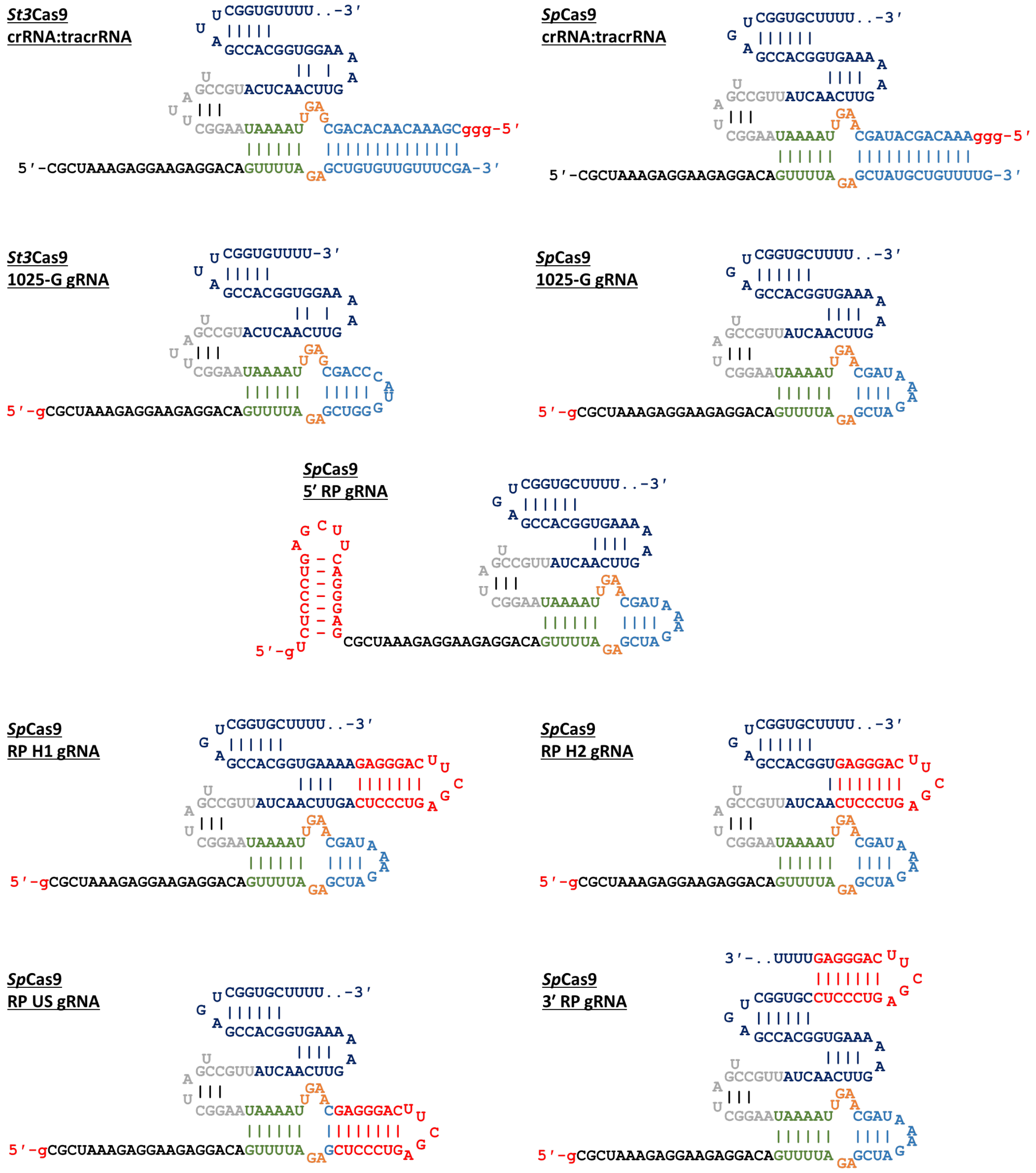

Supplementary Figure 1. Sequences and putative folded structure of crRNA:tracrRNA and gRNA used in this study. Sequences are coloured: red, structural additions; black, Spacer; green, Lower Stem; orange, Bulge; light blue, Upper Stem; Grey, Nexus; dark blue, Hairpins. Red guanines at the 5′ ends are due to *in vitro* transcription. Dotted lines at 3′ ends represent potential variability in the number of uracils due to termination of *in vitro* transcription.

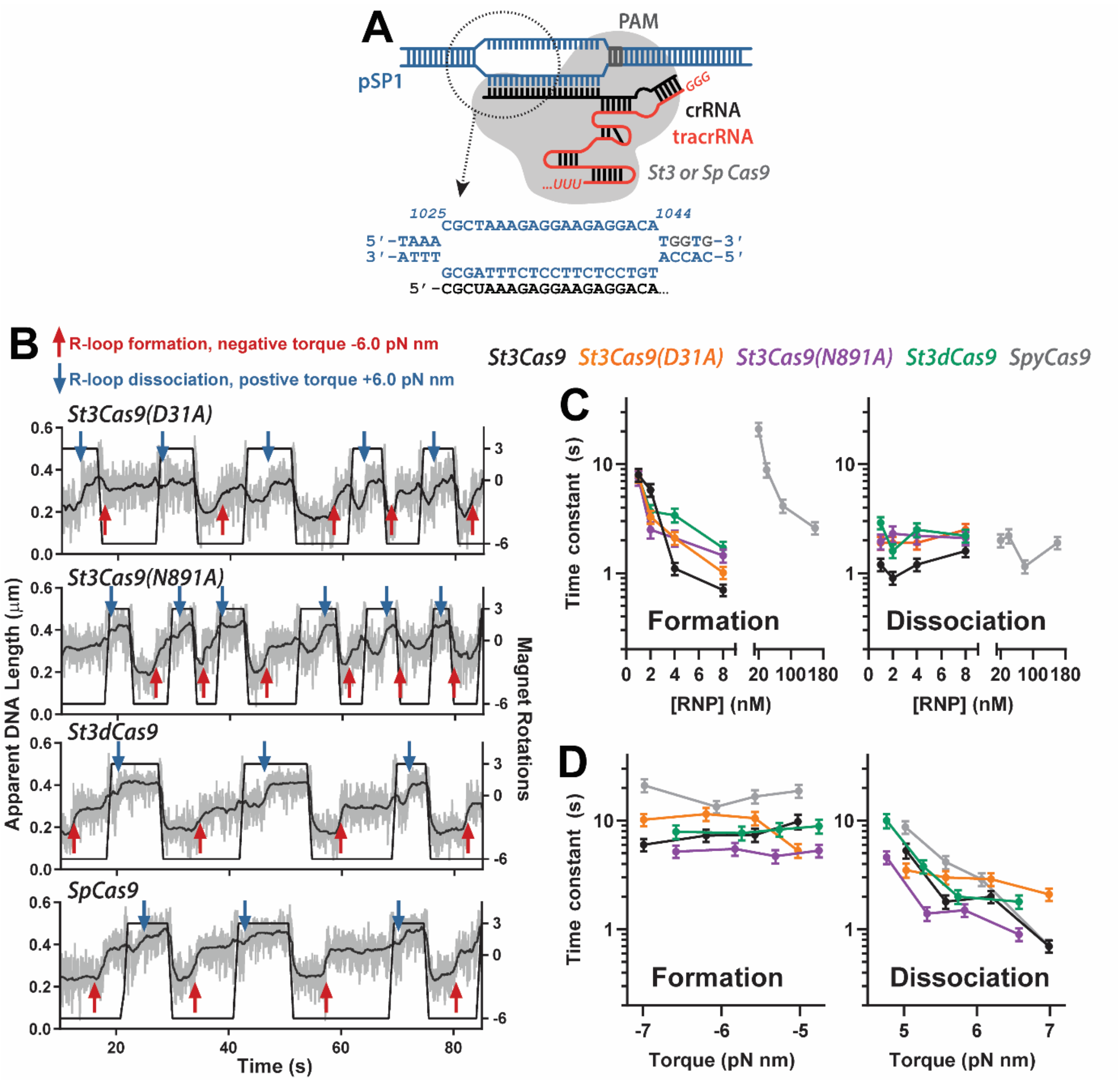

Supplementary Figure 2. R-loop dynamics for *Streptococcus pyogenes* and *Streptococcus thermophilus* DGCC7710 CRISPR3 Cas9 with their respective crRNA:tracrRNA. (**A**) Schematic of R-loop formation. Cas9 (grey) and the cRNA: tracrRNA (black:red) binds the PAM (grey) and forms a 20 bp R-loop at the protospacer sequence at 1025-1044 bp of pSP1 (blue). The sequences of the DNA and crRNA spacer sequence are shown. (**B**) Example MT traces of R-loop cycling (at 10 turns s^{-1}) to measure R-loop formation (red arrows) and dissociation (blue arrows). Raw (grey) and 2 Hz smoothed data (black) are shown. Each enzyme trace represents a different single DNA. (**C**) Mean R-loop formation and dissociation times and standard error (*N* = 31 to 64, Supplementary Table 3) as a function of RNP concentration for WT *St3*Cas9 (black), *St3*Cas9(D31A) (orange), *St3*Cas9(N891A) (purple), *St3*dCas9 (green) and *Sp*Cas9 (grey). *x*-axes are split 0 – 9 nM and 19 – 180 nM. (**D**) Mean R-loop formation and dissociation times and standard error (*N* = 40 to 60, Supplementary Table 3) as a function of torque (enzyme colours as in panel C) using 1 nM RNP for all *Streptococcus thermophilus* RNPs and 10 nM for *Sp*Cas9 RNP. Cumulative probability distributions used to calculate the times in panels C and D are shown in Supplementary Figure 3.

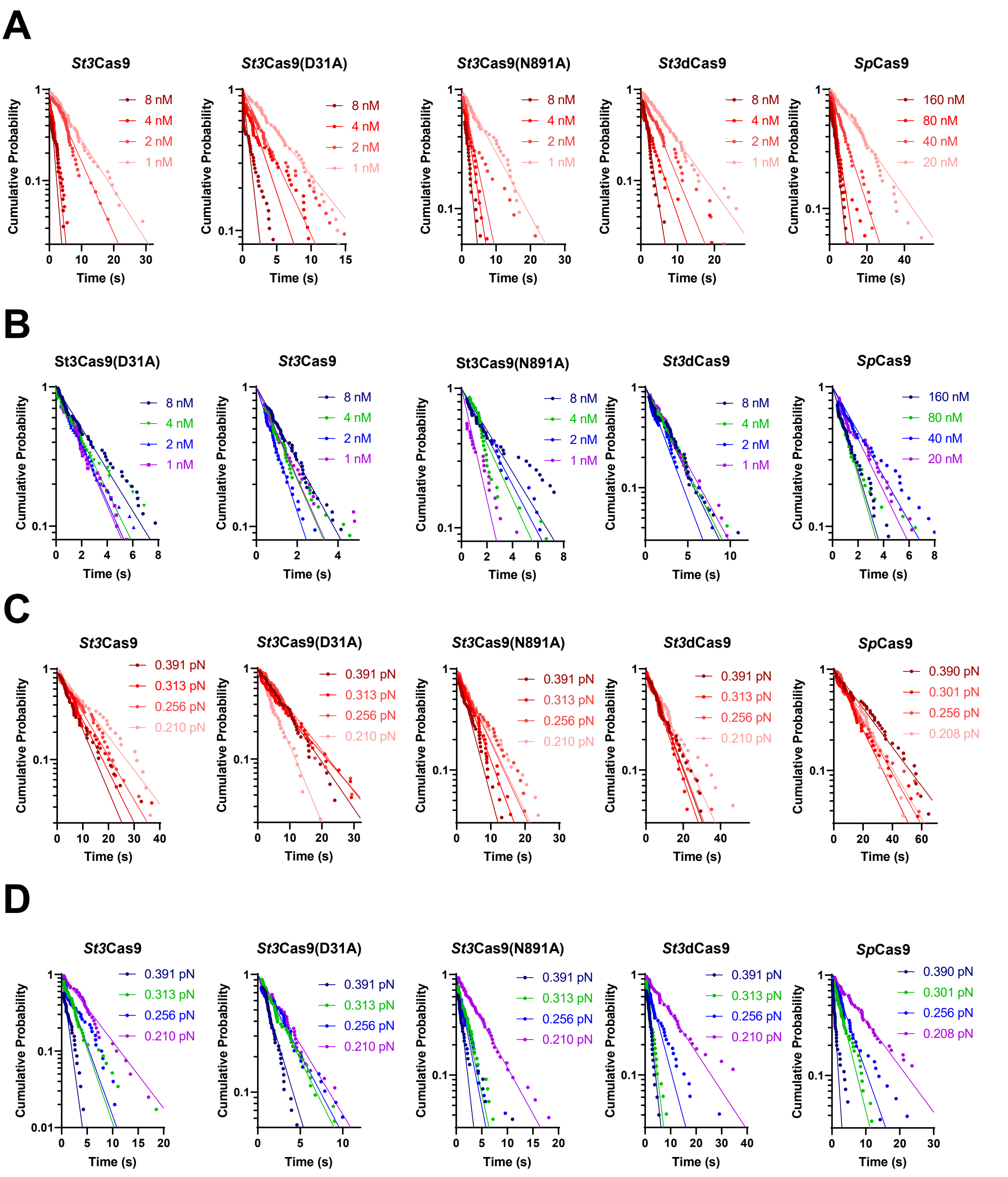

Supplementary Figure 3. Cumulative probability distributions for R-loop formation and dissociation events with Cas9 and crRNA:tracrRNA. R-loop cycling in a magnetic tweezers assay (Figure 1C) was used to analyse the R-loop dynamics for WT *St3*Cas9, the nicking mutants *St3*Cas9(D31A) and *St3*Cas9(N891A), the nuclease dead mutant *St3*dCas9, and WT *Sp*Cas9 (Supplementary Figure 2B). Data were fitted (solid lines) to a single exponential decay apart from those stated in Supplementary Table 3. (**A, B**) R-loop formation (panel A) and dissociation (panel B) times as a function RNP concentration (Cas9:crRNA:tracrRNA = 1:1:1) at 0.313 pN. (**C, D**) R-loop formation (panel C) and dissociation (panel D) times as a function of applied DNA stretching force with an RNP concentration of 1 nM for *St3*Cas9 variants and 10 nM for *Sp*Cas9 (with Cas9:crRNA:tracrRNA = 1:1:1).

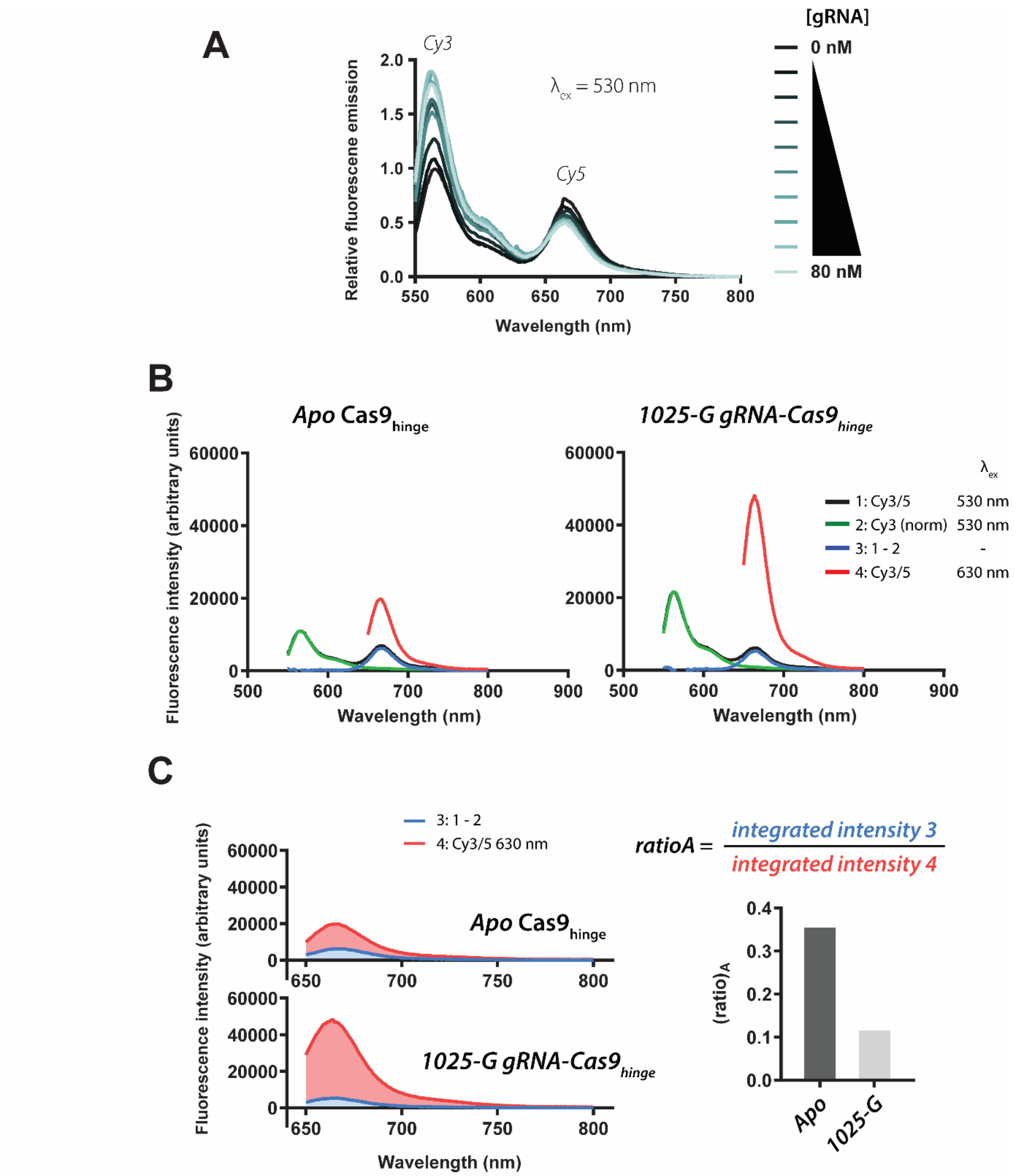

Supplementary Figure 4. FRET measurements of gRNA loading with *Sp*Cas9$_{hinge}$. (**A**) *Sp*Cas9$_{hinge}$ was labelled with Cy3, or with Cy3 and Cy5, at positions 435 and 945 (Materials and Methods). In the Apo state of *Sp*Cas9$_{hinge}$-Cy3/5, there is high acceptor fluorescence. Donor (Cy3) fluorescence rises and acceptor (Cy5) fluorescence falls with increasing [gRNA] when the donor fluorophore is excited at 530 nm. [*Sp*Cas9$_{hinge}$] = 50 nM. (**B, C**) (Ratio)$_A$ can be calculated by dividing the integrated intensity between 650 and 800 nm (scan 3) [calculated as the difference between *Sp*Cas9$_{hinge}$-Cy3/5 (scan 1) and *Sp*Cas9$_{hinge}$-Cy3 (scan 2), excited at 530 nm], by the integrated intensity 4 between 650 and 800 nm (scan 4) [calculated from *Sp*Cas9$_{hinge}$-Cy3/5 directly excited at 630 nm]. Example scans are shown for Apo and 1025-G gRNA bound states.

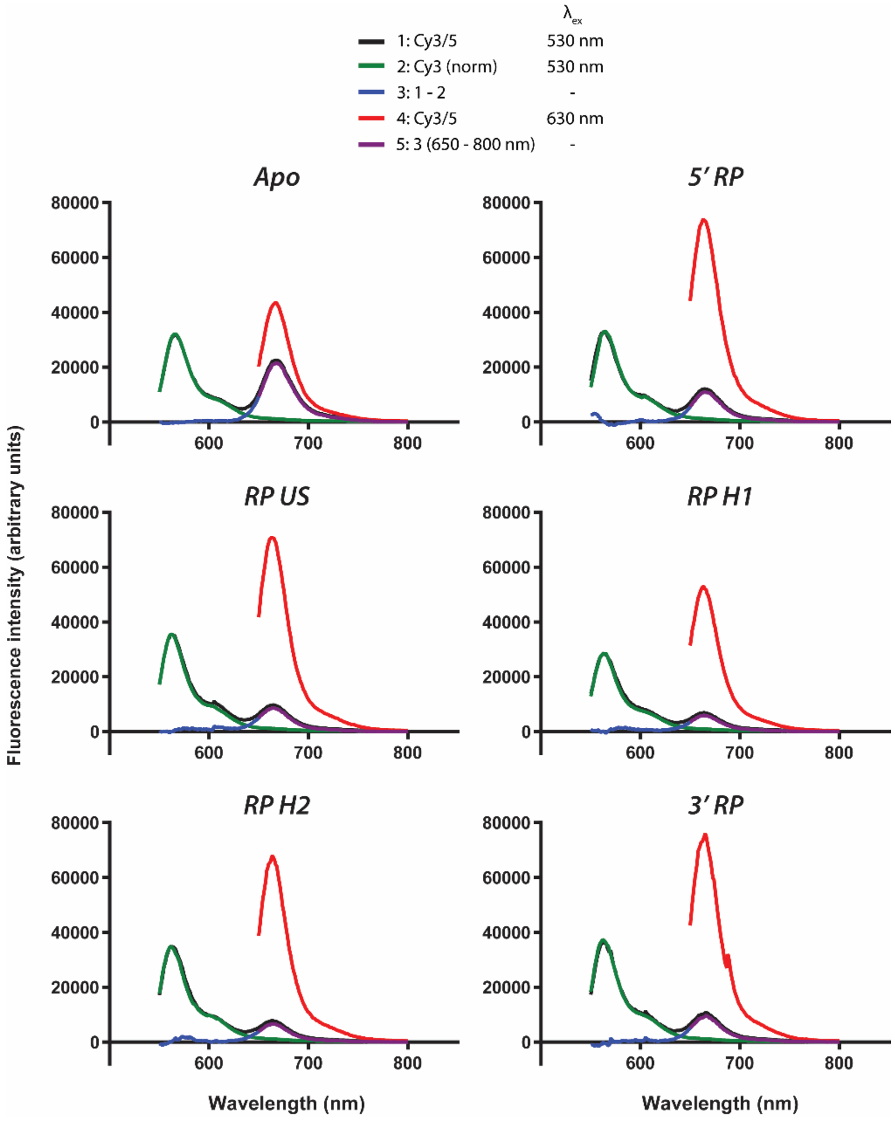

Supplementary Figure 5. Example FRET measurements of loading of *Sp*Cas9$_{hinge}$ with RP-modified gRNAs as indicated.

A

```
Method RK4
STARTTIME = 0
STOPTIME = 300                                          these values changed to actual
DT = 0.02                                               values for each run
d/dt (CCC) = -ka*CCC - kini*CCC
d/dt (ucCCC) = kini*CCC
d/dt (OC) = ka*CCC - kb*OC - kini2*OC -ka*OC1
d/dt (OC1) = -ka*OC1 - kini*OC1
d/dt (ucOC) = kini2*OC + kini*OC1
d/dt (LIN) = kbOC
Tot CCC = CCC + ucCCC                                   uncut OC/CCC allows for reaction
Tot OC = OC + OC1 + ucOC                                not going to completion.
Init CCC = 95
init LIN = 1
init OC1 = 4
init OC = 0
init ucCCC = 0
init ucOC = 0
ka = 0.10                                               these values allowed to be fit
kb = 0.10                                               by software
kini = 0.02
kini2 = 0.02
```

B

```
METHOD RK4
STARTTIME = 0
STOPTIME = 300                                          these values changed to actual
DT = 0.02                                               values for each run
d/dt (CCCC)= -kIN*CCCC
d/dt (CCC) = kIN*CCCC - ka*CCC - kini*CCC
d/dt (ucCCC) = kini*CCC
d/dt (OCC1) = -kIN*OCC1
d/dt (OC1) = kIN*OCC1 - ka*OC1 - kini*OC1
d/dt (OC) = ka*CCC + ka*OC1 - kb*OC - kini2*OC
d/dt (ucOC) = kini2*OC + kini*OC1
d/dt (LIN) = kb*OC
TotCCC = CCCC + CCC + ucCCC                             uncut OC/CCC allows for reaction
TotOC = OCC1 + OC1 + OC + ucOC                          not going to completion.
init CCCC = 95
init CCC = 0
init LIN = 1
init OCC1 = 4
init OC1 = 0
init OC = 0
init ucCCC = 0
init ucOC = 0
ka = 0.14
kb = 0.10                                               these values allowed to be fit
kini = 0.02                                             by the software
kini2 = 0.02
kIN = 0.01
```

Supplementary Figure 6. DNA cleavage models used for numerical integration with Berkeley Madonna. (**A**) Standard A-B-C dsDNA cleavage model that allows for incomplete cleavage. (**B**) Modified A'-A-B-C dsDNA cleavage model that allows for an R-loop formation step prior to cleavage, and for incomplete cleavage.

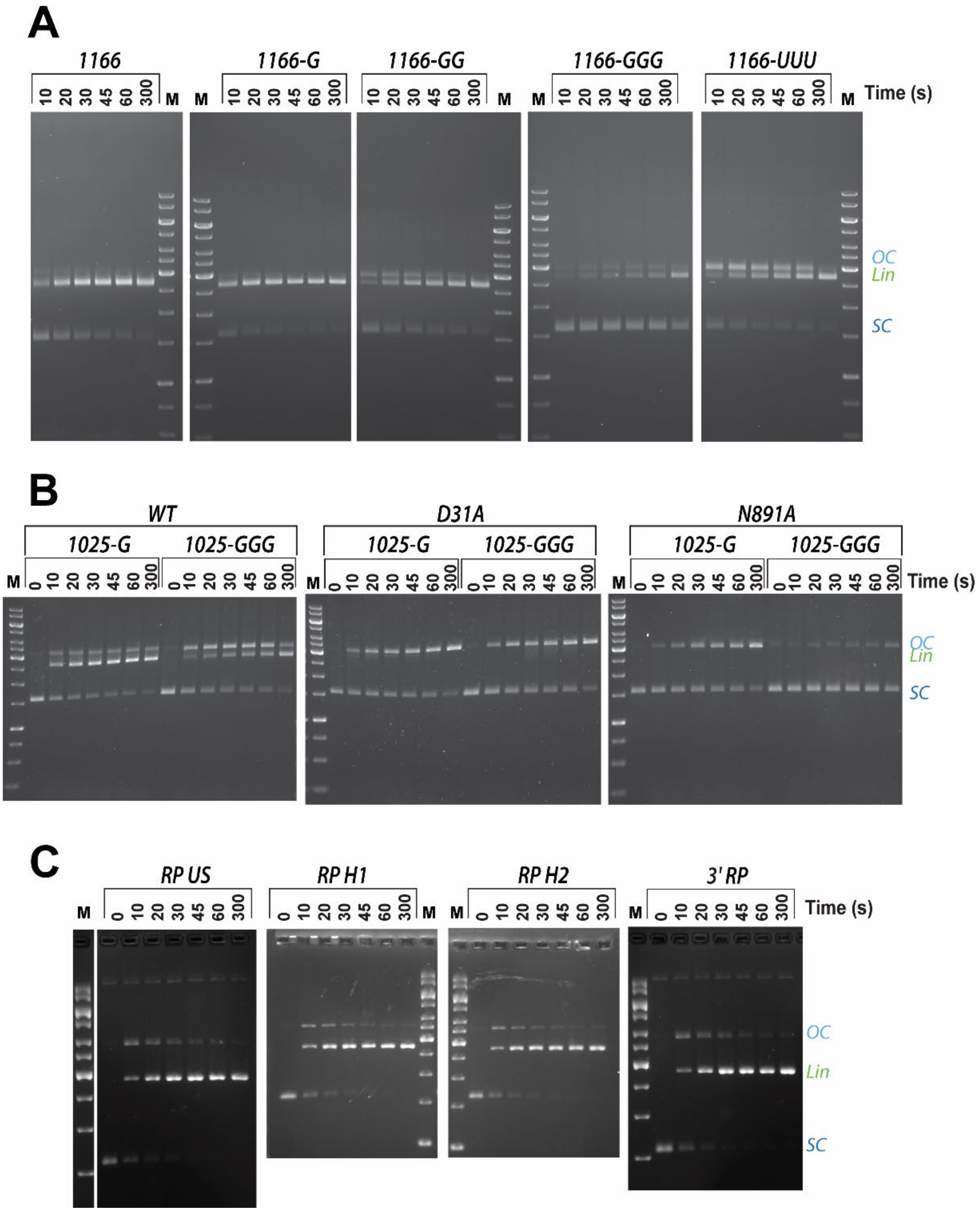

Supplementary Figure 7. Example agarose gels of DNA cleavage assays using pSP1 and either *Sp*Cas9 or *St3*Cas9 with gRNAs as indicated. M is the marker lane with bands (kbp): 10, 8.0, 6.0, 5.0, 4.0, 3.5, 3.0, 2.5, 2.0, 1.5, 1.0, 0.75, 0.5. Gels were run at different V/cm for different times, so the relative separation of the supercoiled (SC), nicked (OC) and linear (LIN) species differs. (**A**) DNA cleavage by *Sp*Cas9 using gRNA with unpaired 5′ modified gRNAs targeted to the 1147-1166 protospacer of pSP1 (Figure 3B). (**B**) DNA cleavage by *St3*Cas9 WT or nicking mutants using gRNA with unpaired 5′ modified gRNAs targeted to the 1025-1044 protospacer of pSP1 (Figure 4). (**C**) DNA cleavage by *Sp*Cas9 using RP-modified gRNAs targeted to the 1025-1044 protospacer of pSP1 (Figure 6A). N.B. The 5′ RP gels are presented in Figure 6B.

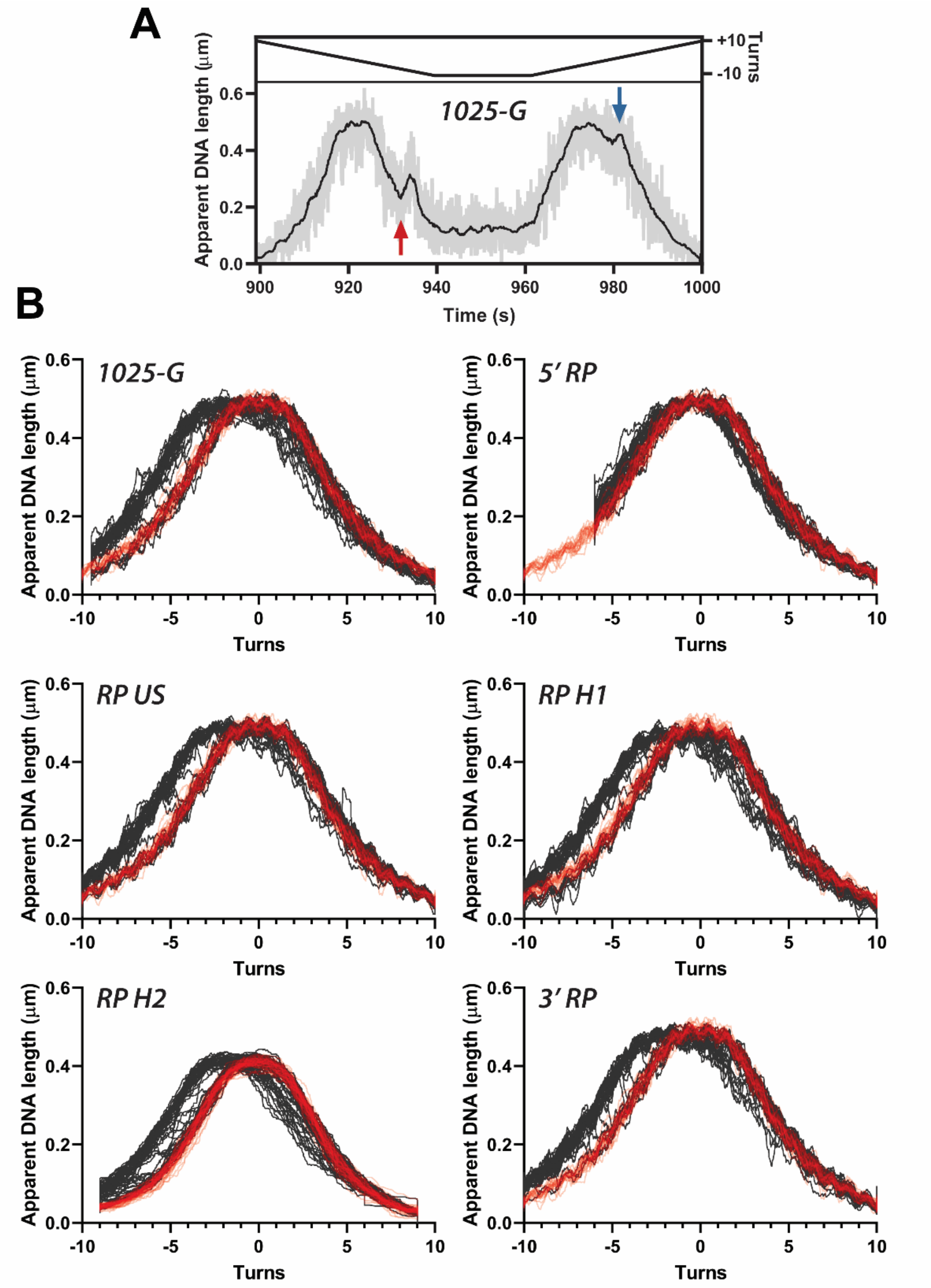

Supplementary Figure 8. R-loop cycling experiments in the MT assay. (**A**) Example of an R-loop cycling experiment at slow rotation speed (1 turn s^{-1}, 0.3 pN) in the presence of 1 nM *Sp*Cas9:1025-G gRNA. Raw DNA length recorded at 60 Hz (grey), data smoothed by a 2 Hz median filter (black). Events marked are R-loop formation at negative torque (red arrow) and dissociation at positive torque (blue arrow). The cycling between +10 and -10 turns is then repeated. (**B**) Overlay of repeated R-loop cycles (data smoothed by 2 Hz median filter) in the absence of Cas9 (red, *N* = 15), or in the presence of 1 nM *Sp*Cas9:gRNA as indicated (dark grey, *N* = 18-20). Rotation curve shifts (Figure 5E) were calculated by comparing the return parts of the curves where an R-loop is present from -6 to -4 turns. The RP H2 data was measured on a different DNA to the other gRNAs. The 5′ RP data was cycled between +10 and -6 to avoid surface interactions of the R-loop captured state.

Synthetic RNA	Sequence (5' - 3') – spacer sequence in lowercase.	Supplier
*St3*Cas9 crRNA 1025	cgcuaaagaggaagaggacaGUUUUAGAGCUGUGUUGUUUCG	Metabion
*Sp*Cas9 crRNA 1025	cgcuaaagaggaagaggacaGUUUUAGAGCUAUGCUGUUUUG	Metabion
*Sp*Cas9 1166	gcucacucauuaggcaccccGUUUUAGAGCUAGAAAUAGCAAGUUAAAAUAAGG CUAGUCCGUUAUCAACUUGAAAAAGUGGCACCGAGUCGGUGCUUUU	IDT Ultramer
*Sp*Cas9 1166-G	GgcucacucauuaggcaccccGUUUUAGAGCUAGAAAUAGCAAGUUAAAAUAAG GCUAGUCCGUUAUCAACUUGAAAAAGUGGCACCGAGUCGGUGCUUUU	IDT Ultramer
*Sp*Cas9 1166-GG	GGgcucacucauuaggcaccccGUUUUAGAGCUAGAAAUAGCAAGUUAAAAUAA GGCUAGUCCGUUAUCAACUUGAAAAAGUGGCACCGAGUCGGUGCUUUU	IDT Ultramer
*Sp*Cas9 1166-GGG	GGGgcucacucauuaggcaccccGUUUUAGAGCUAGAAAUAGCAAGUUAAAAUA AGGCUAGUCCGUUAUCAACUUGAAAAAGUGGCACCGAGUCGGUGCUUUU	IDT Ultramer
*Sp*Cas9 1166-UUU	UUUgcucacucauuaggcaccccGUUUUAGAGCUAGAAAUAGCAAGUUAAAAUA AGGCUAGUCCGUUAUCAACUUGAAAAAGUGGCACCGAGUCGGUGCUUUU	IDT ultramer
*St3*Cas9 1025-G	GcgcuaaagaggaagaggacaGUUUUAGAGCUGGGUACCCAGCGAGUUAAAAUA AGGCUUAGUCCGUACUCAACUUGAAAAGGUGGCACCGAUUCGGUGUUUU	IDT ultramer
*St3*Cas9 1025-GGG	GGGcgcuaaagaggaagaggacaGUUUUAGAGCUGGGUACCCAGCGAGUUAAAA UAAGGCUUAGUCCGUACUCAACUUGAAAAGGUGGCACCGAUUCGGUGUUUU	IDT ultramer

Supplementary table 1. Synthetic crRNA and gRNA.

**DNA substrates for IVT**			
**RNA**	**Plasmid**	**DNA sequence (5′ - 3′) – spacer in lowercase.**	**Primers pairs for PCR**
*St3*Cas9 tracrRNA	pCRISPR3	CGAAACAACACAGCGAGTTAAAATAAGGCTTAGTCCGTACTCAACTTGAAAAGGTGGCACCGATTCGGTGTTTTT	TK-4 T7 Rev, TK-52 T7
*Sp*Cas9 tracrRNA	pX330	GTTTTAGAGCTAGAAATAGCAAGTTAAAATAAGGCTAGTCCGTTATCAACTTGAAAGTTTTAGAGCTAGAAATAGCAAGTAAGTGGCACCGAGTCG GTGCTTTTTT	TK-109 T7 Rev, TK-120 T7
1025-G	pD1301i-SP1	cgctaaagaggaagaggacaGTTTTAGAGCTAGAAATAGCAAGTTAAAATAAGGCTAGTCCGTTATCAACTTGAAAAAGTGGCACCGAGTCGGTGCTTTTT T	oli#GM RP T7 (G)<br>oli#GM T7 Rev
1025-GG	pD1301i-SP1	As 1025-G	oli#GM RP T7 (GG)<br>oli#GM T7 Rev
1025-GGG	pD1301i-SP1	As 1025-G	oli#GM RP T7 (GGG)<br>oli#GM T7 Rev
5′ RP	pEX-A2-g#GM SP1gRNA 5′ RP	TCTCCCTGAGCTTCAGGGAGcgctaaagaggaagaggacaGTTTTAGAGCTAGAAATAGCAAGTTAAAATAAGGCTAGTCCGTTATCAACTTGAAAAAGT GGCACCGAGTCGGTGCTTTTTT	oli#GM RP T7 (G)<br>oli#GM T7 Rev
5′ RP U_1	pEX 5′ RP U	TCTCCCTGAGCTTCAGGGAGTcgctaaagaggaagaggacaGTTTTAGAGCTAGAAATAGCAAGTTAAAATAAGGCTAGTCCGTTATCAACTTGAAAAAGT GGCACCGAGTCGGTGCTTTTTT	oli#GM RP T7 (G)<br>oli#GM T7 Rev
5′ RP U_2	pEX 5′ RP 2U	TCTCCCTGAGCTTCAGGGAGTTcgctaaagaggaagaggacaGTTTTAGAGCTAGAAATAGCAAGTTAAAATAAGGCTAGTCCGTTATCAACTTGAAAAAG TGGCACCGAGTCGGTGCTTTTTT	oli#GM RP T7 (G)<br>oli#GM T7 Rev
5′ RP U_4	pEX 5′ RP 4U	TCTCCCTGAGCTTCAGGGAGTTTTcgctaaagaggaagaggacaGTTTTAGAGCTAGAAATAGCAAGTTAAAATAAGGCTAGTCCGTTATCAACTTGAAAA AGTGGCACCGAGTCGGTGCTTTTTT	oli#GM RP T7 (G)<br>oli#GM T7 Rev
5′ RP U_8	pEX 5′ RP 8U	TCTCCCTGAGCTTCAGGGAGTTTTTTTTcgctaaagaggaagaggacaGTTTTAGAGCTAGAAATAGCAAGTTAAAATAAGGCTAGTCCGTTATCAACTTGA AAAAGTGGCACCGAGTCGGTGCTTTTTT	oli#GM RP T7 (G)<br>oli#GM T7 Rev
RP US	pEX-A2-g#GM SP1gRNA US RP	cgctaaagaggaagaggacaGTTTTAGAGCTCCCTGAGCTTCAGGGAGCAAGTTAAAATAAGGCTAGTCCGTTATCAACTTGAAAAAGTGGCACCGAGTC GGTGCTTTTTT	oli#GM SP1 T7 (G)<br>oli#GM T7 Rev
RP H1	pEX-A2-g#GM SP1gRNA H RP_1	cgctaaagaggaagaggacaGTTTTAGAGCTAGAAATAGCAAGTTAAAATAAGGCTAGTCCGTTATCAACTTGACTCCCTGAGCTTCAGGGAGAAAAGTG GCACCGAGTCGGTGCTTTTTT	oli#GM SP1 T7 (G)<br>oli#GM T7 Rev
RP H2	pEX-A2-g#GM SP1gRNA H RP_2	cgctaaagaggaagaggacaGTTTTAGAGCTAGAAATAGCAAGTTAAAATAAGGCTAGTCCGTTATCAACTCCCTGAGCTTCAGGGAGTGGCACCGAGTC GGTGCTTTTTT	oli#GM SP1 T7 (G)<br>oli#GM T7 Rev
RP 3′	pEX-A2-g#GM SP1gRNA 3′ RP	cgctaaagaggaagaggacaGTTTTAGAGCTAGAAATAGCAAGTTAAAATAAGGCTAGTCCGTTATCAACTTGAAAAAGTGGCACCGAGTCGGTGCTCTC CCTGAGCTTCAGGGAGTTTTTT	oli#GM SP1 T7 (G)<br>oli#GM 3'RP T7 Rev

**Synthetic DNA primers**		
	**DNA sequence (5′ - 3′) – T7 promoter in bold.**	Supplier
TK-4 T7 Rev	AAAAACACCGAATCGGTGCCAC	Metabion
TK-52 T7	**TAATACGACTCACTATAGGG**CGAAACAACACAGCGAGTTAAAATAAGG	Metabion
TK-109 T7 Rev	AAAAAGCACCGACTCGGTGCCAC	Metabion
TK-120 T7	**TAATACGACTCACTATAGGG**CAAAACAGCATAGCAAGTTAAAATAAGGCTAGTCCG	Metabion
oli#GM SP1 T7 (G)	GACA**CTAATACGACTCACTATAG**CGCTAAAGAGGAAGAGG	IDT
oli#GM SP1 T7 (GG)	GACA**CTAATACGACTCACTATAGG**CGCTAAAGAGGAAGAGG	IDT
oli#GM SP1 T7 (GG)	GACA**CTAATACGACTCACTATAGGG**CGCTAAAGAGGAAGAGG	IDT
oli#GM RP T7 (G)	GACA**CTAATACGACTCACTATAG**TCTCCCTGAGCTTCAGG	IDT
oli#GM T7 Rev	AAAAGCACCGACTCGGTGCC	IDT
oli#GM 3'RP T7 Rev	AAAACTCCCTGAAGCTCAGG	IDT

Supplementary Table 2. DNA sequences of plasmids and synthetic DNA oligonucleotides used for PCR to make dsDNA substrates for *in vitro* transcription of gRNA and tracrRNA.

*Sp*Cas9	[Cas9] nM	*Exponential fitted data formation events*						*Exponential fitted data dissociation events*					
		Torque pN nm	$\tau_{formation}$ (s)				*N*	Torque pN nm	$\tau_{dissociation}$ (s)				*N*
**cr:tracr**	20*	-6.0	100%	21.02 ± 2.89			53	+6.0	100%	2.00 ± 0.27			53
**cr:tracr**	40*	-6.0	100%	8.89 ± 1.34			44	+6.0	100%	2.25 ± 0.33			44
**cr:tracr**	80*	-6.0	100%	4.15 ± 0.58			51	+6.0	100%	1.15 ± 0.16			51
**cr:tracr**	160*	-6.0	100%	2.59 ± 0.34			59	+6.0	100%	1.23 ± 0.24			59
**cr:tracr**	20*	-5.0	100%	18.73 ± 2.50			56	+5.0	100%	8.73 ± 1.16			56
**cr:tracr**	20*	-5.5	100%	16.66 ± 2.33			51	+5.5	100%	4.15 ± 0.58			51
**cr:tracr**	20*	-6.0	100%	13.41 ± 1.77			57	+6.0	100%	2.89 ± 0.38			57
**cr:tracr**	20*	-6.9	100%	21.07 ± 2.86			54	+6.9	100%	0.71 ± 0.09			55
**1G**	10#	-6.2	100%	3.02 ± 0.51			36	+6.2	100%	1.93 ± 0.32			36
**2G**	10#	-6.2	100%	1.50 ± 0.23			43	+6.2	100%	2.85 ± 0.43			43
**3G**	10#	-6.2	100%	2.21 ± 0.34			42	+6.2	100%	7.09 ± 1.09			42
**5′ RP**	10#	-6.2	61%	36.10 ± 8.28	39%	3.57 ± 0.82	19	+6.2	36%	0.07 ± 0.02 s	64%	8.72 ± 2.00	19
**US RP**	10#	-6.2	100%	18.68 ± 3.74			25	+6.2	100%	0.40 ± 0.08 s			25
**H1 RP**	10#	-6.2	100%	10.90 ± 2.73			16	+6.2	100%	0.64 ± 0.16 s			16
**H2 RP**	10#	-6.2	100%	5.28 ± 1.04			26	+6.2	54%	0.39 ± 0.08 s	46%	5.91 ± 1.16	26
**3′ RP**	10#	-6.2	100%	16.81 ± 3.86			19	+6.2	100%	0.61 ± 0.14s			19

*St3*Cas9	[Cas9] nM	*Exponential fitted data formation events*						*Exponential fitted data dissociation events*					
		Torque pN nm	$\tau_{formation}$ (s)				*N*	Torque pN nm	$\tau_{dissociation}$ (s)				*N*
**cr:tracr**	1	-6.0	100%	8.03 ± 1.07			56	+6.0	100%	1.16 ± 0.15			55
**cr:tracr**	2	-6.0	100%	5.82 ± 0.80			53	+6.0	100%	0.93 ± 0.12			53
**cr:tracr**	4	-6.0	100%	1.28 ± 0.17			58	+6.0	100%	1.19 ± 0.15			58
**cr:tracr**	8	-6.0	100%	0.68 ± 0.08			64	+6.0	100%	1.58 ± 0.20			63
**cr:tracr**	1	-5.0	100%	9.76 ± 1.53			41	+5.0	100%	5.33 ± 0.83			40
**cr:tracr**	1	-5.5	100%	7.41 ± 1.03			51	+5.5	100%	1.83 ± 0.25			50
**cr:tracr**	1	-6.1	100%	7.33 ± 0.94			60	+6.1	100%	2.02 ± 0.26			58
**cr:tracr**	1	-7.0	100%	6.02 ± 0.79			58	+7.0	100%	0.68 ± 0.09			58

*St3*Cas9 D31A	[Cas9] nM	*Exponential fitted data formation events*						*Exponential fitted data dissociation events*					
		Torque pN nm	$\tau_{formation}$ (s)				*N*	Torque pN nm	$\tau_{dissociation}$ (s)				*N*
**cr:tracr**	1	-6.0	100%	7.89 ± 1.11			50	+6.0	100%	1.94 ± 0.27			50
**cr:tracr**	2	-6.0	100%	3.31 ± 0.45			53	+6.0	100%	1.94 ± 0.26			51
**cr:tracr**	4	-6.0	100%	2.07 ± 0.28			57	+6.0	100%	1.87 ± 0.25			57
**cr:tracr**	8	-6.0	100%	1.01 ± 0.13			58	+6.0	100%	2.48 ± 0.33			57
**cr:tracr**	1	-5.0	100%	5.29 ± 0.78			46	+5.0	100%	3.47 ± 0.51			46
**cr:tracr**	1	-5.5	100%	10.45 ± 1.50			49	+5.5	100%	3.03 ± 0.42			49
**cr:tracr**	1	-6.1	75%	11.49 ± 1.58	25%	1.24 ± 0.17	53	+6.1	100%	2.93 ± 0.40			53
**cr:tracr**	1	-7.0	100%	8.90 ± 1.17			57	+7.0	100%	2.10 ± 0.28			57

*St3*Cas9 N891A	[Cas9] nM	*Exponential fitted data formation events*						*Exponential fitted data dissociation events*					
		Torque pN nm	$\tau_{formation}$ (s)				*N*	Torque pN nm	$\tau_{dissociation}$ (s)				*N*
**cr:tracr**	1	-6.0	100%	7.48 ± 1.14			43	+6.0	100%	0.78 ± 0.11			43
**cr:tracr**	2	-6.0	100%	2.54 ± 0.43			34	+6.0	100%	2.34 ± 0.39			31
**cr:tracr**	4	-6.0	100%	2.10 ± 0.35			36	+6.0	100%	2.22 ± 0.37			36
**cr:tracr**	8	-6.0	100%	1.45 ± 0.20			51	+6.0	100%	2.08 ± 0.29			50
**cr:tracr**	1	-4.7	100%	5.29 ± 0.71			56	+4.7	100%	4.56 ± 0.61			53
**cr:tracr**	1	-5.3	100%	4.67 ± 0.66			51	+5.3	100%	1.34 ± 0.20			48
**cr:tracr**	1	-5.8	100%	5.53 ± 0.74			55	+5.8	100%	1.45 ± 0.20			55
**cr:tracr**	1	-6.5	79%	5.24 ± 0.68	21%	0.31 ± 0.04	59	+6.5	100%	0.85 ± 0.11			55

*St3*dCas9	[Cas9] nM	*Exponential fitted data formation events*						*Exponential fitted data dissociation events*					
		Torque pN nm	$\tau_{formation}$ (s)				*N*	Torque pN nm	$\tau_{dissociation}$ (s)				*N*
**cr:tracr**	1	-6.0	100%	7.79 ± 1.00			61	+6.0	100%	2.85 ± 0.37			60
**cr:tracr**	2	-6.0	100%	3.71 ± 0.52			50	+6.0	100%	1.62 ± 0.23			50
**cr:tracr**	4	-6.0	64%	3.45 ± 0.49	36%	0.24 ± 0.03	48	+6.0	100%	2.49 ± 0.36			48
**cr:tracr**	8	+6.0	100%	1.70 ± 0.23			57	+6.0	100%	2.17 ± 0.29			56
**cr:tracr**	1	+4.7	100%	8.87 ± 1.32			45	+4.7	100%	10.07 ± 1.49			44
**cr:tracr**	1	+5.2	100%	8.31 ± 1.19			49	+5.2	100%	3.76 ± 0.54			49
**cr:tracr**	1	+5.7	100%	7.69 ± 1.10			49	+5.7	100%	21.97 ± 0.28			49
**cr:tracr**	1	+6.5	100%	7.89 ± 1.11			51	+6.5	100%	1.84 ± 0.25			50

Supplementary Table 3. Time constants from the fits to the R-loop formation and dissociation times measured from the magnetic tweezers assay (Figures 3D, 5G, 5H, and Supplementary Figure 3). Data plotted in Figures 2D, 2E, 3E, and 5I. 100% indicates single exponential, two percentages indicate the amplitudes of the fast and slow phases of a double exponential. **Sp*Cas9 preparation 2014-09-23. #New England Biolabs *Sp*Cas9 M0386. Errors are standard errors based on *N* values.

(± SD)	$k_{formation}$ (s^{-1})	k_a (s^{-1})	k_b (s^{-1})	k_{ini} (s^{-1})	k_{ini2} (s^{-1})	N
**Figure 3H**						
**1025+G**	N/A	0.0484 ± 0.0068	0.10545 ± 0.00501	0.0211 ± 0.0107	0.0091 ± 0.0013	4
**1025+GG**	N/A	0.0214 ± 0.0006	0.00392 ± 0.00041	0.0067 ± 0.0059	0.0007 ± 0.0002	4
**1025+GGG**	N/A	0.0216 ± 0.0014	0.00098 ± 0.00019	0.0115 ± 0.0033	0.0005 ± 0.0002	3
**Figure 6C**						
**1025+G**	N/A	0.0987 ± 0.0756	0.1041 ± 0.0132	0.0047 ± 0.0017	0.0062 ± 0.0008	3
**RP US**	N/A	0.0883 ±0.0085	0.1281 ± 0.0180	0.0067 ± 0.0012	0.0100 ± 0.0037	3
**RP H1**	N/A	0.1156 ± 0.0177	0.1565 ± 0.0314	0.0057 ± 0.0027	0.0107 ± 0.0039	3
**RP H2**	N/A	0.0871 ± 0.0075	0.1603 ± 0.0271	0.0067 ± 0.0020	0.0098 ± 0.0032	3
**3' RP**	N/A	0.0990 ± 0.0056	0.1239 ± 0.0107	0.0045 ± 0.0022	0.0078 ± 0.0019	3
**Figure 4F**						
**1166**	N/A	0.071 ± 0.031	0.722 ± 0.430	0.007 ± 0.004	0.106 ± .068	3
**1166+G**	N/A	n.d.	n.d.	n.d.	n.d.	3
**1166+GG**	N/A	0.089 ± 0.041	0.042 ± 0.015	0.018 ± 0.011	0.009 ± 0.001	3
**1166+GGG**	N/A	0.009 ± 0.003	0.021 ± 0.003	0.007 ± 0.001	0.009 ± 0.004	3
**1166+GGG (2)**	0.017 ± 0.004	0.14 fixed	0.048 ± 0015	0.114 ± 0.05	0.015 ± 0.007	3
**1166+UUU**	N/A	0.129 ± 0.037	0.019 ± 0.002	0.019 ± .007	0.005 ± 0.002	3

Supplementary Table 4. Apparent rate constants from the numerical integration fits of the DNA cleavage data in Figures 3F, 4B, 4E and 6A to the models in Supplementary Figure 6. Each repeat was fitted separately, and the fitted parameters averaged. Errors are standard deviation (SD).

**Tukey's multiple comparisons test**	**Mean Diff.**	**95.00% CI of diff.**	**Significant?**	**Summary**	**Adjusted P Value**
1G vs. **5' RP**	0.9900	0.6968 to 1.283	Yes	****	<0.0001
1G vs. RP US	-0.05000	-0.3353 to 0.2353	No	ns	0.9958
1G vs. RP H1	0.1200	-0.1691 to 0.4091	No	ns	0.8341
1G vs. RP H2	-0.2900	-0.5753 to -0.004660	Yes	*	0.0440
1G vs. 3' RP	-0.06000	-0.3453 to 0.2253	No	ns	0.9901
**5' RP** vs. RP US	-1.040	-1.333 to -0.7468	Yes	****	<0.0001
**5' RP** vs. RP H1	-0.8700	-1.167 to -0.5732	Yes	****	<0.0001
**5' RP** vs. RP H2	-1.280	-1.573 to -0.9868	Yes	****	<0.0001
**5' RP** vs. 3' RP	-1.050	-1.343 to -0.7568	Yes	****	<0.0001
RP US vs. RP H1	0.1700	-0.1191 to 0.4591	No	ns	0.5311
RP US vs. RP H2	-0.2400	-0.5253 to 0.04534	No	ns	0.1519
RP US vs. 3' RP	-0.01000	-0.2953 to 0.2753	No	ns	>0.9999
RP H1 vs. RP H2	-0.4100	-0.6991 to -0.1209	Yes	**	0.0010
RP H1 vs. 3' RP	-0.1800	-0.4691 to 0.1091	No	ns	0.4663
RP H2 vs. 3' RP	0.2300	-0.05534 to 0.5153	No	ns	0.1880

Supplementary Table 5. Ordinary one-way ANOVA test using Tukey's multiple comparison of the data from Figure 5E, 5F, calculated using GraphPad Prism (GraphPad Software, San Diego, CA).
